# Mesenchymal stem cells injected into carotid artery to target focal brain injury home to perivascular space

**DOI:** 10.7150/thno.43169

**Published:** 2020-05-17

**Authors:** Anna Andrzejewska, Sylwia Dabrowska, Blazej Nowak, Piotr Walczak, Barbara Lukomska, Miroslaw Janowski

**Affiliations:** 1 NeuroRepair Department, Mossakowski Medical Research Centre, PAS, Warsaw, Poland; 2 Department of Neurosurgery, Central Clinical Hospital of Ministry of the Interior and Administration, Warsaw, Poland; 3 Center for Advanced Imaging Research, Department of Diagnostic Radiology and Nuclear Medicine, University of Maryland Marlene and Stewart Greenebaum Comprehensive Cancer Center, University of Maryland, Baltimore, MD, USA; 4 Tumor Immunology and Immunotherapy Program, University of Maryland Marlene and Stewart Greenebaum Comprehensive Cancer Center, University of Maryland, Baltimore, MD, USA

**Keywords:** mesenchymal stem cells, integrin VLA4 (α4β1), mRNA cell transfection, brain injury, intra-arterial delivery, perivascular space

## Abstract

**Rationale**: The groundbreaking discovery of mesenchymal stem cells (MSCs) with their multifaceted benefits led to their widespread application in experimental medicine, including neurology. Efficient delivery of MSCs to damaged regions of the central nervous system may be a critical factor in determining outcome. Integrin VLA-4 (α4β1) coded by ITGA4 and ITGB1 genes is an adhesion molecule expressed by leukocytes, which is responsible for initiation of their diapedesis through cell docking to the inflamed vessel wall expressing VCAM1 receptor. This function of VLA-4 has been recapitulated in neural stem cells and glial progenitors. Thus, it was prudent to investigate this tool as a vehicle driving extravasation of MSCs. Since MSCs naturally express ITGB1 subunit, we decided to supplement them with ITGA4 only. The purpose of our current study is to investigate the eventual fate of IA delivered ITGA4 engineered and naive MSCs.

**Methods**: mRNA-ITGA4 transfected and naive MSCs were injected to right internal carotid artery of rats with focal brain injury. Through next three days MSC presence in animals' brain was navigated by magnetic resonance imaging. Transplanted cell location relative to the brain blood vessels and host immunological reaction were analyzed post-mortem by immunohistochemistry. The chemotaxis of modified and naive MSCs was additionally examined in *in vitro* transwell migration assay.

**Results**: Both naïve and ITGA4-overexpressing cells remained inside the vascular lumen over the first two days after IA infusion. On the third day, 39% of mRNA-ITGA4 modified and 51% naïve MSCs homed to perivascular space in the injury region (p=NS). The gradual decrease of both naive and mRNA-ITGA4 transfected hBM-MSCs in the rat brain was observed. mRNA-ITGA4 transfected MSCs appeared to be more vulnerable to phagocytosis than naïve cells. Moreover, *in vitro* study revealed that homogenate from the injured brain repels migration of MSCs, corroborating the incomplete extravasation observed *in vivo*.

**Conclusions**: In summary, IA transplanted MSCs are capable of homing to the perivascular space, an integral part of neurovascular unit, which might contribute to the replacement of injured pericytes, a critical element facilitating restoration of CNS function. The mRNA-ITGA4 transfection improves cell docking to vessel but this net benefit vanishes over the next two days due to fast clearance from cerebral vessels of the majority of transplanted cells, regardless of their engineering status. The drawbacks of mRNA-ITGA4 transfection become apparent on day 3 post transplantation due to the lower survival and higher vulnerability to host immune attack.

## Introduction

Focal damage to the brain, such as that which occurs in stroke or traumatic brain injury, is the most frequent cause of long-term, serious disability and lacks effective treatment; thus a search for new therapies is warranted. The discovery of stem cells has created a unique opportunity for brain repair. Mesenchymal stem cells (MSCs) have become the workhorse of regenerative medicine 1. Multiple studies revealed positive effects of MSCs in treatment of stroke 2-4 and traumatic brain injury 5. However, the mechanisms mediating the therapeutic effects of MSCs are largely unknown. While there is growing evidence of the therapeutic effects of MSC-derived extracellular vesicles (EVs) 6,7, MSCs are still the mainstay of regenerative attempts of the central nervous system.

The method of exogenous cell administration has a tremendous impact on their biodistribution as well as the efficacy of their engraftment at a desired destination. Systemic administration of cells by intravenous injection is currently the most commonly used technique in cell transplantation. While straightforward to perform, it is plagued by very low homing in the brain. In this context, the intra-arterial (IA) route emerges as a very attractive alternative due to the ability to precisely control the destination of cells in a minimally invasive fashion 8. This technique potentially allows for reducing the number of cells necessary to achieve their therapeutic dose as well as avoiding the risk of their deposition in an undesirable organ, such as the lung or liver. Nevertheless, the engraftment efficacy remains insufficient and the fate of MSCs in the CNS requires further studies.

Regardless of the route (intravenous or IA), the transplanted cells have to extravasate to produce the benefit from their presence in the central nervous system. Immune cells perfected this task through ligand-receptor interactions. VLA-4 - VCAM1 axis is a well-known contributor to diapedesis of leukocytes. VLA-4 is expressed on the surface of immune cells while VCAM1 is expressed on the endothelium. This axis was employed first to prove the potential role of ligand-receptor interaction in trafficking of neural stem cells to the central nervous system (CNS) 9. Then, we revealed that engineering of glial progenitors to induce the expression of VLA4 *via* DNA plasmid transfection is instrumental for their docking 10 and diapedesis 11 across VCAM1-positive, inflamed endothelium. VLA-4 is composed of two subunits: ITGA4 and ITGB1. Since ITGB1 is abundantly expressed by MSCs, we decided to supplement the cells with induction of ITGA4 expression only. We have previously shown that MSCs are relatively resistant to DNA plasmid transfection, while prone to mRNA-based cell engineering 12. Since mRNA-based technique for induction of gene expression is virus-free, thus clinically preferable, we used it to complement endogenously expressed ITGB1 with externally introduced ITGA4 *via* mRNA transfection. Moreover, we revealed that mRNA-ITGA4 engineering increases docking of MSCs *in vitro* and *in vivo* 13. Therefore, it was prudent to extend the observation time to 3 days to better understand the value of cell engineering not only on the process of docking but also on diapedesis and ultimate extravasation of MSCs. While long-term expression of the transgene may be desirable in many applications, in our studies the short-term protein expression characteristic for introduced mRNA is what is required for our study. Diapedesis is a relatively fast process 14, and transient overexpression of VLA-4 is sufficient for its completion. Permanent overexpression of receptors involved in cell migration and diapedesis can cause problems associated with transplant safety and disturbances of stemness instead of providing increased therapeutic efficacy.

There are a variety of stroke models used in experimental studies. Initial MRI studies revealed that an evolution of neuropathological changes after intracerebral administration of ouabain closely resembles ischemic stroke 15. Indeed, ouabain is an inhibitor of Na+/K+-ATPase, an enzyme responsible for the majority of energetic demand of excitable cells. Thus, its blockade mimics a stroke-induced shortage of energy. Our extensive studies further validated the value of this model of reproducing a stroke-induced cascade of events 16. The advantage of ouabain model of stroke is its minimal invasiveness, which prevents induction of artificial surgery-related systemic inflammation, as well as a precise control of the size and location of the lesion through stereotactic ouabain administration. We have previously reported positive effects of intra-arterial cell transplantation in ouabain model of stroke 17, as well as the real-time MRI monitoring of IA transplanted cells 18.

Our current study focuses on investigating the eventual fate of IA transplanted mRNA-ITGA4 modified or naive MSCs in this focal brain injury model, with a focus on their engraftment, specifically the niche where these cells settle inside the recipient's brain.

## Methods

### Human bone marrow-derived mesenchymal stem cell (hBM-MSCs) culture and labelling

hBM-MSCs (Lonza Inc. Walkersville, MD, USA) were thawed according to manufacturer's instructions and plated onto 75 cm^2^ flasks at a density 1x10^4^/cm^2^ and grown in MSCBM medium (Lonza Inc. Walkersville, MD, USA) supplemented with 10% MCGS, L-glutamine and gentamycin, at standard environmental conditions (21 % oxygen, 5 % CO_2_, 95 % humidity and 37 °C), with culture medium changed twice a week. hBM-MSCs were passaged after reaching a 70-80 % confluence, which corresponded to intervals of 5-7 days. The cells from passages 5-6 were used in experiments. Molday ION (superparamagnetic iron oxide nanoparticles (SPIO) conjugated with rhodamine B™; BioPAL) was used for cell labelling as we previously described 19. Briefly, hBM-MSCs were incubated over an 18-hour time period with Molday ION at a dose of 20 μg Fe/ml followed by triple washing with PBS and placement of cells in MSCBM medium until experiments were performed.

### Transfection of hBM-MSCs using ITGA4-mRNA

Molday ION-labeled hBM-MSCs were transfected with ITGA4 mRNA as we previously described 12. Briefly, the cDNA for ITGA4 gene was inserted into the pSP72 vector (P2191-Promega) and served as a template for *in vitro* production of mRNA capped with an anti-reverse-cap-analogue (ARCA) using the mMessage mMachine ® T7 Ultra Kit (AM1345, Ambion). Then, the mRNA-ITGA4 (0.94μg/ml) was mixed with Lipofectamine 2000 to form complexes, which were incubated with cells over 4 hours followed by triple washing with PBS and placement of cells in MSCBM medium for 4-6 hours to allow for ITGA4 protein production prior to experiments.

### The use of animals

All procedures were performed in accordance with the Guidelines for the Care and Use of Laboratory Animals adopted by the Institutional Animal Care and Use Committee of the Mossakowski Medical Research Centre, Warsaw, Poland and as recommended by ARRIVE guidelines (Animal Research: Reporting *In Vivo* Experiments). Procedures were approved by the IV Local Ethics Committee in Warsaw (agreement no 17/2012). Thirty-six adult male Wistar rats approximately 7-8 weeks in age (250 g weight) were housed in cages with a 12-hour light-dark cycle with free access to food and water under standard humidity and temperature. All experiments were designed in order to minimize the number of animals used and their suffering.

### Ethical approval

All procedures complied with EU guidelines for the use of animals in research and were approved by the regulations of IV Local Ethics Committee Animal Experiments in Warsaw (agreement no 17/2012).

### Focal brain injury

Stroke-like focal brain injury model was performed as we previously described 16. Briefly, under general anesthesia, a burr hole was placed in the skull and a needle (length 15 mm, gauge 33) connected to a 10 μl syringe (Hamilton, Switzerland) was inserted into brain at coordinates: A 0.5; L 3.8; D 4.7 mm. Then, 1 μl of 5 mmol ouabain solution (Sigma, Poland) was injected over 1 minute using a microinfusion pump (Stoelting, USA) and five minutes later the needle was withdrawn and the skin was closed with a suture. After the procedure, each animal was injected with an antibiotic (Baytril; Bayer; 0.4 mg/ml) and an analgesic (Rycarfa; Krka; 5 mg/ml).

### IA transplantation of hBM-MSCs

All animals were randomly given numerical equivalents and experimenters performing further analysis were not aware of the animal's assignment to the experimental group. Animals received mRNA-ITGA4 transfected or naïve hBM-MSCs. Cells were transplanted intra-arterially 48 hours after induction of focal brain injury as we previously described 18. Briefly, animals were anaesthetized with 2% isoflurane and after visualization of the common carotid artery (CCA), external carotid artery (ECA), and internal carotid artery (ICA), the occipital artery branching off the ECA was closed by coagulation, and the pterygopalatine artery branching off the ICA was ligated along with proximal segments of the ECA and CCA. Then, the proximal part of ICA was clipped (FT 180T, Aesculap, Center Valley, PA, USA) to prevent backflow while the incision of CCA was performed distal to the ligature, and the catheter was introduced into the CCA, pushed into the ICA until reaching the closure by the vascular clip, and stabilized by tightening the suture on the artery. Then the clip was removed and the animal was transferred to the gantry of the MR scanner. Next 5x10^5^ Molday ION-labeled mRNA ITGA-engineered or control (naïve) hBM-MSCs suspended in 1 ml of PBS were injected at a rate of 0.2 ml/min.

### Magnetic resonance imaging (MRI) and quantification

Administration of hBM-MSCs was recorded by a 7T Biospec 70/30 MR scanner (Bruker), with a transmit cylindrical radiofrequency coil (8.6cm inner diameter) and a rat brain-dedicated, receive-only array coil (2x2 elements) positioned over the animal's head. Directly before and after transplantation, animals were imaged using a T2 sequence (RARE, TR=4000ms, TE=58.5ms, TA=2m8s, FOV=2.69/2.35, MTX=256/128) to detect brain injury and with a T2* sequence (FLASH, TR=500ms, TE=5ms, FA=40deg, TA=48s, FOV=2.69/2.35, MTX=256/128) to detect injected cells. The procedure was repeated over the next three days after cells transplantation. Three T2* scans for each animal were further analyzed in Sentinels Application Platform (SNAP; Brockmann Consult). Four separate regions of interest (ROI) were outlined within each picture: ROI1: area from the left edge of rat's body to the middle of brain section not including the left brain hemisphere; ROI2: area of the left brain hemisphere; ROI3: area from the right edge of rat's body to the middle of the brain section not including the right brain hemisphere; ROI4: area of the right brain hemisphere. Pixel values together with corresponding X and Y coordinates were collected for all four ROIs separately and saved as numerical data. Data was imported to Microsoft Office Excel spreadsheet (Microsoft). All pixel values within the same Y plane were clustered and numbered according to X coordinate. For ROI1 and ROI2, pixel values were numbered from minimal to maximal X values, whereas for ROI3 and ROI4, values were numbered from maximal to minimal X values. This provided a unique label for each pixel in Y group of a particular ROI. Next, the division of pixel values with identical label from ROI1 to ROI3 were calculated - hereafter referred to as ROI1/ROI3 rate. Also, the division of pixel values with identical label from ROI2 to ROI4 were calculated - hereafter referred to as ROI2/ROI4 rate. All values of ROI1/ROI3 rate lower than 0.01 and higher than 5.00 were rejected from further analysis. Data such as mean value and standard deviation (SD) were calculated for ROI1/ROI3 within the same Y plane. For each Y plane, the mean value + 2 SD was calculated and described as final Y threshold value. All previously calculated ROI2/ROI4 rates were compared to final Y threshold value from the same Y plane. If ROI2/ROI4 rate was lower than or equal to final Y threshold value, data was assigned to group hereafter referred to as group A. Consequently, if ROI2/ROI4 rate was higher than final Y threshold, it was assigned to group hereafter referred to as group B. The quantity of all obtained ROI2/ROI4 rates was calculated and recognized as 100% of dataset elements. Each ROI2/ROI4 rate was recognized as one element of data set independently from its value. The percentage of values assigned to group B in the whole data set was determined. The percentage of group B indicated the degree of occupation of transplanted cells within the injured hemisphere.

### Immunohistochemistry (IHC)

The animals were euthanized and decapitated, and the brains were immediately removed, snap-frozen and cryo-cut to 20 µm thick slices. Upon thawing, fixation was carried out by incubation of slices for 15 minutes in 4% PFA. Then the brain sections were permeabilized and blocked in 0.25% Triton X-100, 10% goat serum and 1% bovine serum albumin (BSA) in PBS solution for 90 min at a room temperature. Primary antibodies: mouse IgG1 anti-CD44, -ED1, -Claudin-5, rabbit polyclonal anti-laminin, or rabbit polyclonal anti-Claudin 5 and chicken IgY anti-Map2 were incubated overnight with brain sections at 4oC. After thorough washing, the secondary antibodies: goat anti-mouse IgG1 Alexa Fluor 488, goat anti-mouse IgG1 Alexa Fluor 546, goat anti-rabbit IgG Alexa Fluor 633 and goat anti-chicken IgY Alexa Fluor 488 were incubated for 60 min in the dark at room temperature. The cell nuclei were counterstained with 5 μM Hoechst 33258 solution (Sigma) for 5 minutes. Slices were closed using a fluorescence mounting medium (Dako). The confocal microscope (Zeiss LSM 780) was used for image acquisition and Z-stack projection creation and processing. Cell Observer SD system was used to perform stitching of tiles and employed to provide high-resolution images of the entire right brain section. The number of cells positive for a particular marker was determined within all cells exhibiting fluorescence in the spectral range of excitation wavelength 546 nm specific for the Molday ION, which was used for hBM-MSCs labelling prior to intra-arterial administration. Cell counting was done manually in the ZEN 2012 blue (Zeiss) program. The confocal microscope studies were performed in Laboratory of Advanced Microscopy Techniques, Mossakowski Medical Research Centre, PAS.

### Cell migration assay

The migration capability of mRNA-ITGA4 transfected and naïve hBM-MSCs was assessed using transwell chambers (Corning). The inserts containing polycarbonate membranes with 8 μm pores were coated with type I collagen (Gibco) at a concentration of 50 μg/ml and incubated for 60 minutes at room temperature followed by three washes with PBS. The subset of membranes was additionally coated with VCAM1 protein (10 μg/ml) by their overnight incubation at 4°C in protein solution. Then the chambers were triple washed with PBS prior to their use in experiments. Opti-MEM® I Reduced Serum Medium (Life Technologies) with 0.1 % BSA or homogenate obtained from 48-hour old focal rat brain injury by ouabain as described above at a protein concentration 0.5 mg/ml were placed in the lower compartment of the chamber. ITGA4-mRNA transfected or naïve hBM-MSCs labelled with Molday ION suspended in 100 μl Opti-MEM® I Reduced Serum Medium at a dose 1x10^4^ per membrane were placed in the upper compartment of the chamber. After 24 hours of incubation (37°C, 5% CO_2_, humidity 95%), membranes were washed with PBS. The fixation of the hBM-MSCs remaining on the membrane was made by 15 minutes of incubation in a 4% PFA solution. Additionally, the nuclei were counterstained by 5 minutes of incubation with 5 μM Hoechst 33258 solution. Cells present on the upper surface of the membrane were removed mechanically using cotton swabs. The images of entire membranes were obtained by stitching tiles acquired by the Axio Observer Z.1 microscope and analysed in the ZEN 2012 blue system. The cell nuclei were used to count the total number of hBM-MSCs present on the lower surface of the membrane.

### Statistical analysis

To determine the statistical significance, the type III fixed effects test was used, and the LMS method was applied for comparison. Box plots present the distribution of data for the test value in the compared populations. The length of the presented bars is equal to the quadrant (Q1-Q3) data, the whiskers indicate the minimum and maximum values, the line within the bar determines the median, while the arithmetic means are shown as squares and outliers are presented as circles. * p <0.05, ** p <0.01 *, *** p <0.001 (n = 6). Data implemented in the text indicate the mean value ± standard derivation.

## Results

### Biodistribution of intra-arterially-transplanted hBM-MSCs based on serial MRI

**Figure 1:**
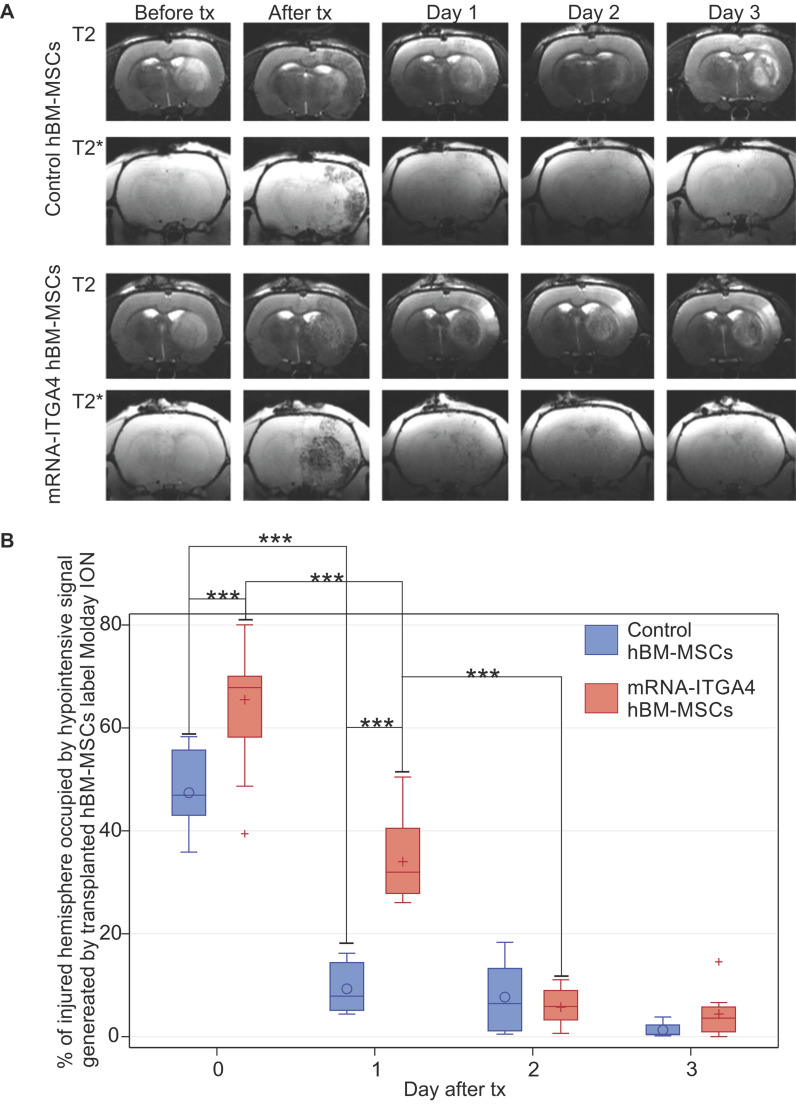
Evaluation of the presence of IA transplanted mRNA-ITGA4 transfected or control (naïve) hBM-MSCs in the rat brain subjected to focal brain damage using MRI scan assessment. A) mRNA-ITGA4 transfected and control hBM-MSCs labelled with Molday ION were visible in MRI in T2 and T2* scans up to three days after transplantation(tx) B) The box-plot graph shows the percentage of right hemisphere occupied by hypo-intensive signal generated by transplanted mRNA-ITGA4 transfected (red boxes) or Control hBM-MSCs (violet boxes). The type III fixed effects test was used to determine statistical significance, and the LMS method was applied to compare between groups and time points. Box charts present the dispersion and the shape of the data distribution for the test value in the compared populations. The length of the bars is equal to the quarter range (Q1-Q3) of the data, the tips of the mustaches indicate the minimum and maximum values, the line inside of the bar determines the median, while the circle/plus the arithmetic mean, the outliers are presented in the form of circles/pluses; *p <0.05, **p <0.01, ***p <0.001 (n=6)

Infusion of hBM-MSC to the ipsilateral ICA resulted in cells being captured within the infarcted area (Figure 1A, Figure S1). The transplanted cells were visible in MRI scans as hypo-intensive signal due to their previous labeling with magnetic contrast - nanoparticles of iron oxide conjugated with fluorescent dye Rhodamine B. The mRNA-ITGA4 engineering of hBM-MSCs increased their initial brain uptake (Figure 1B), and the result is in line with our previous report 13. After one day, there was a significant reduction of naïve (p<0.001) and engineered hBM-MSCs (p<0.001) (Figure 1B). Notably, the rate of transplanted cell clearance from the cerebral vasculature was equal for both engineered and naïve MSCs at two initial time points (0 and 24 hours). The reduction of transplanted cell presence in the brain continued, and at the end of the second day after transplantation the signal of transplanted cells was barely present, while the difference between both types of cells vanished and became stable between the second and the third day of observation after transplantation. Therefore, no observations were carried out beyond that time point (Figure 1B).

### Microscopic detection of intra-arterially delivered hBM-MSCs

**Figure 2:**
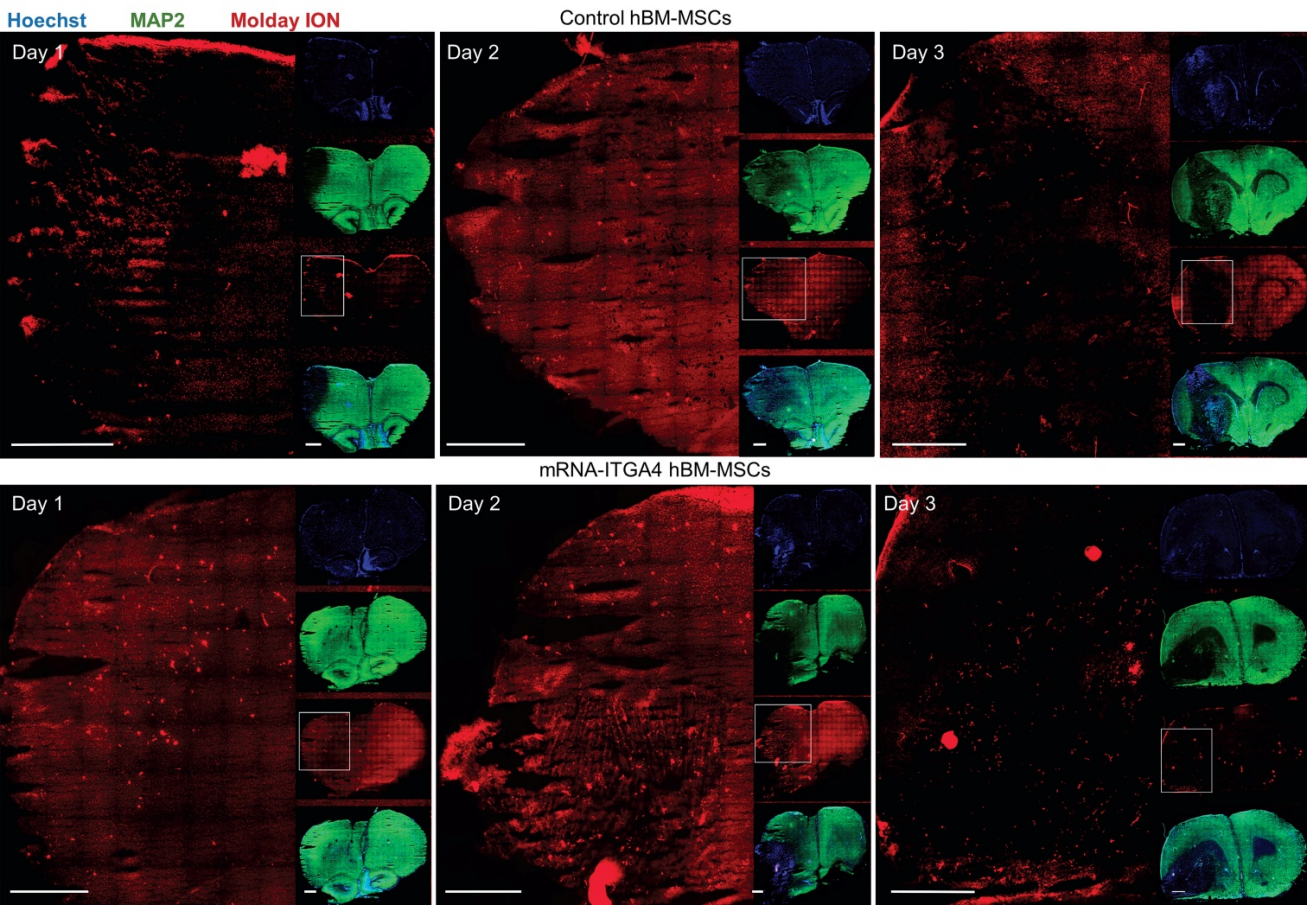
Tile scans of immunohistochemically stained rat brain tissues after focal brain injury showing IA transplanted hBM-MSCs in many different cross section planes on the first, second and the third day after cell infusion. Both control hBM-MSCs as well as mRNA-ITGA4 transfected cells (red) were visible in the injured hemisphere. The Map2 antibody staining (green) was used to display the necrotic penumbra area. Most transplanted cells were observed in the core and the boundary of necrotic penumbra region. The cell nuclei were counterstained with Hoechst 33258 (blue). Scale 1mm.

**Figure 3:**
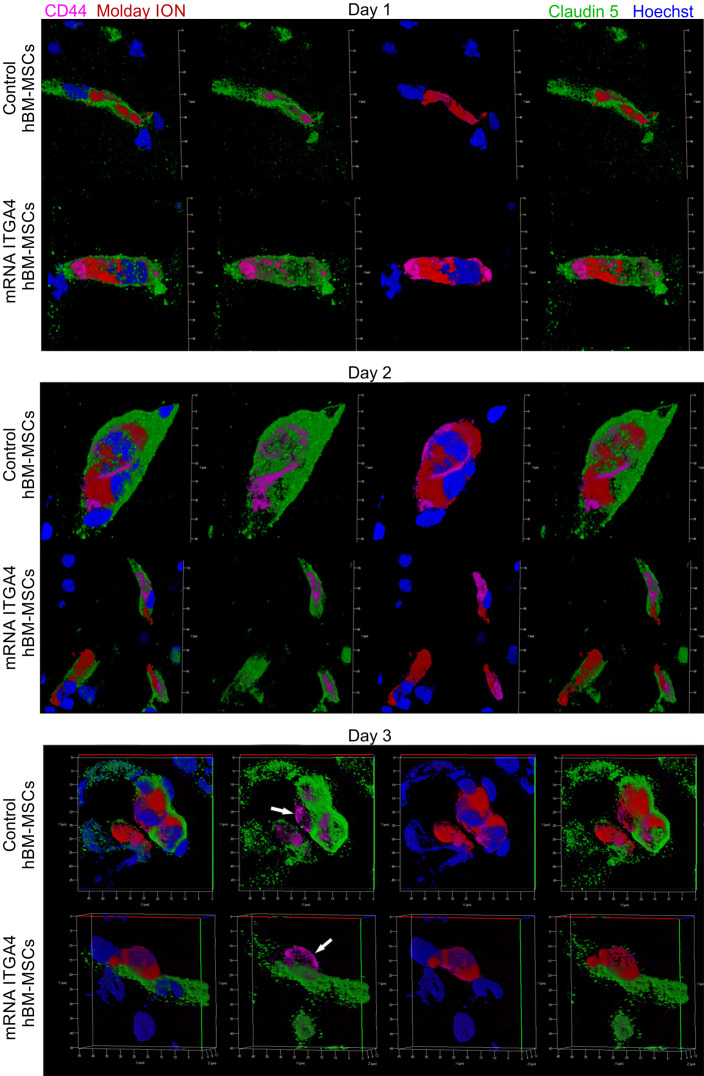
Immunohistochemical analysis of transplanted hBM-MSCs located within the area of focal brain injury in relation the vessel lumen through three days after IA transplantation in rats with focal brain injury. mRNA-ITGA4 transfected and naïve hBM-MSCs were both labelled with Molday ION (red) before IA injection. Transplanted cells were detected in the host brain post-mortem using anti-CD44 antibody (magenta). Both populations of IA infused MSCs (control and mRNA-ITGA4 transfected cells) cells were visible exclusively inside the lumen of cerebral blood vessels on day 1 and 2, interestingly their extravasation was observed on day 3 after IA injection. Endothelial cells were visualized using antibody against Claudine 5 (green). The cell nuclei were counterstained with Hoechst 33258 (blue).

**Figure 4:**
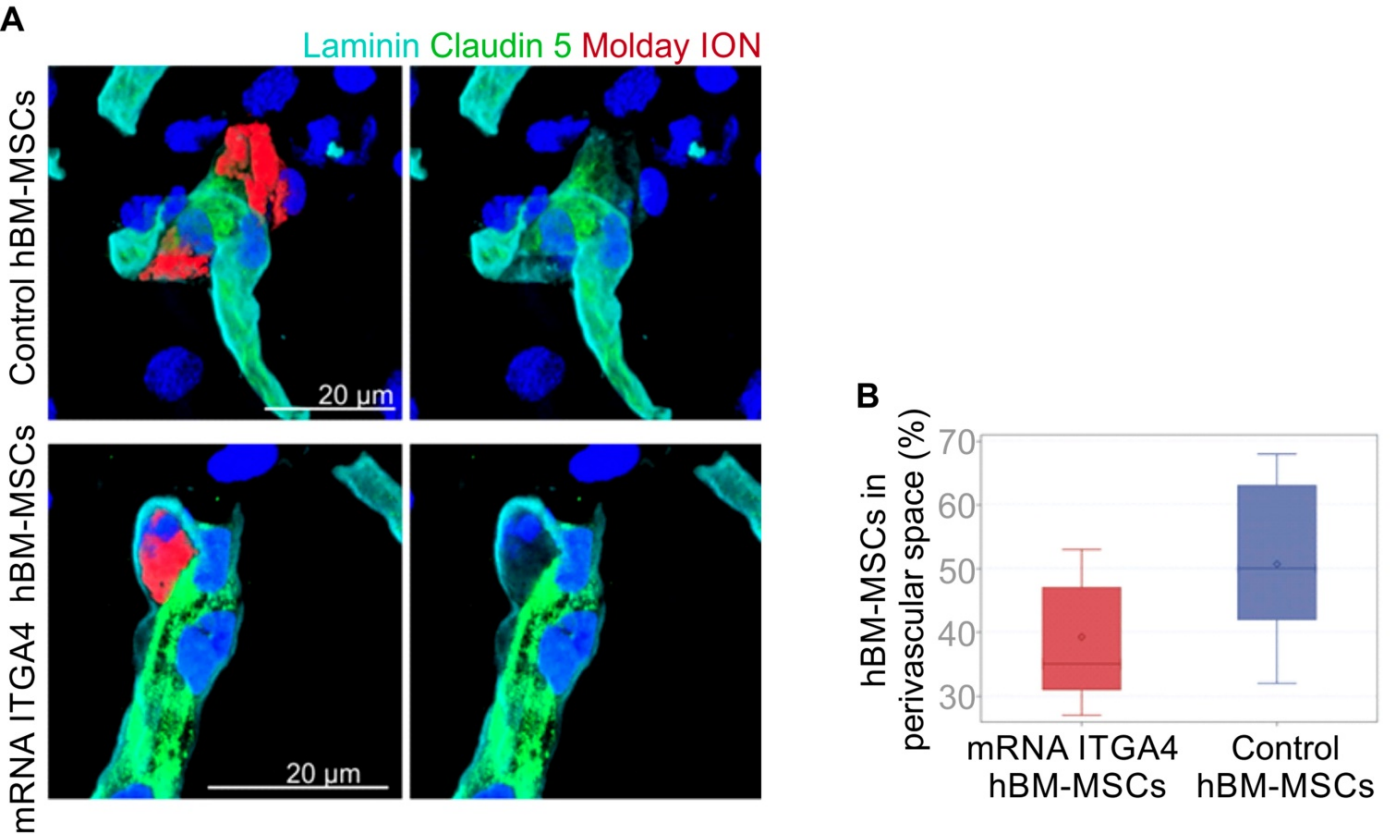
Immunohistochemical analysis of IA injected control or mRNA-ITGA4 transfected hBM-MSC. The presence of transplanted cells was assessed in cerebral blood vessels of the focally injured rat brain on the third day after transplantation. (A) Confocal images revealed Molday ION-labelled hBM-MSCs located between endothelium visualized by staining with Claudin 5 antibody (green) and basal membrane detected by the anti-laminin antibody (turquoise). The cell nuclei were counterstained with Hoechst 33258 (blue). (B) The graph presents the percentage of mRNA-ITGA4 (red boxes) and Control (violet boxes) hBM-MSCs localized in the perivascular space of brain blood vessels on the third day after IA transplantation in rats subjected to the focal brain injury. The type III fixed effects test was apply to determine statistical significance, and the LMS method was used to compare. Box charts present the dispersion and shape of the data distribution for the test value in both tested cell populations. The length of the bars is equal to the quarter range (Q1-Q3) of the data, the tips of the moustaches indicate the minimum and maximum values, the line inside the bar determines the median, while the square the arithmetic mean, the outliers are presented in the form of circles; *p<0.05, **p<0.01, ***p<0.001 (n=6)

IHC detected both mRNA-ITGA4 modified and control (naïve) IA transplanted hBM-MSCs inside the injured hemisphere in many different planes of infarcted brain cross-section (Figure 2, Figure S2). Transplanted cells were visible on the first, second, and third day post transplantation. No transplanted cells were present in the contralateral hemisphere. Most hBM-MSCs were localized in the boundary and the core of necrotic penumbra visualized by MAP-2 antibody staining. All transplanted cells were located inside the vessel lumen for the first and second day as revealed by co-localization of Molday ION and CD44 antigen (mesenchymal stem cell marker) and staining for Claudin 5 (marker of brain endothelial cells tight junctions). On the third day after transplantation, some hBM-MSCs were located outside of the blood vasculature, but they still were adhering to their external walls (Figure 3, Figure S3). The additional staining for laminin revealed that extravasated cells located in the perivascular space limited on one side by endothelial cells, and on the other side by the basal membrane of the blood vessels (Figure 4A). Quantitative analysis of extravasating cells revealed a strong trend towards a higher percentage of naive compared to engineered hBM-MSCs (51±13.25% and 39±10.40%, respectively) present in the perivascular space; however, the difference was not statistically significant (p=0.06) (Figure 4B).

### mRNA-ITGA4 transfected hBM-MSCs are more vulnerable to phagocytosis in vivo

**Figure 5:**
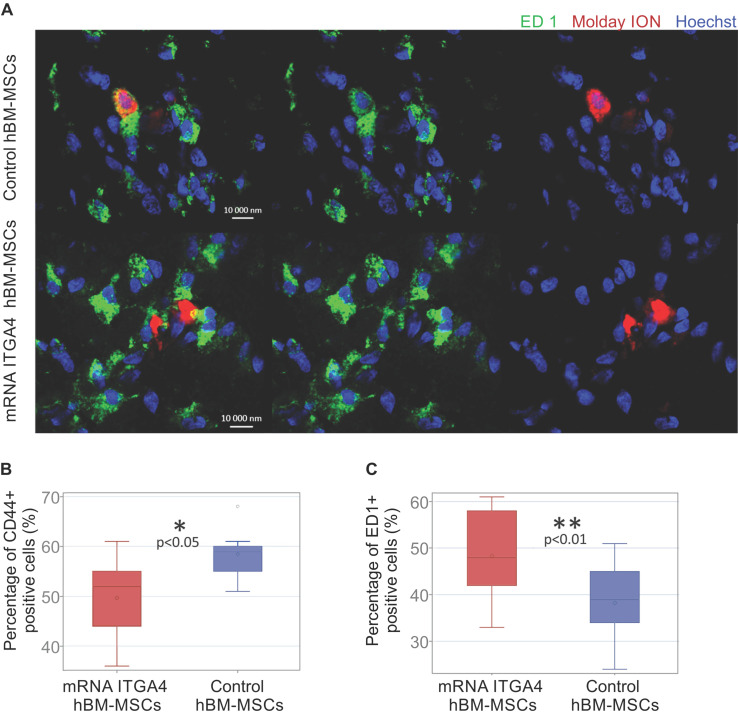
Immunocytochemical characteristics of transplanted hBM-MSCs and rat microglia/macrophages present in the focally injured rat brain on the third day after IA cell injection. (A) Co-localization of transplanted Molday ION positive mRNA-ITGA4 transfected or Control hBM-MSCs (red) with ED1^+^ cells (green) probably points to phagocytosis of the donor cells by microglia/macrophages of the host. The graph presents the percentage of Molday ION positive mRNA-ITGA4 transfected or control hBM-MSCs which co-localised CD44 (B) and ED1 (C) antigen. The type III fixed effects test was used to determine statistical significance, and the LMS method was used to compare. Box charts present the dispersion and the shape of the data distribution for the test value in both cell populations. The length of the bars is equal to the quarter range (Q1-Q3) of the data, the tips of the moustaches indicate the minimum and maximum values, the line inside of the bar determines the median, while the circle the arithmetic mean, the outliers are presented in the form of circles; *p<0.05, ** p<0.01, ***p <0.001 (n=6)

The identity of transplanted hBM-MSCs was confirmed using anti-CD44 staining in recipient brains collected 3 days after cell transplantation (Figure 5A). Antibody against ED1 antigen was used to detect microglia. Among all Molday ION-labelled hBM-MSCs 49.67 ± 8.25% of mRNA-ITGA4 transfected cells and 58.44 ± 4.57% of naïve cells showed the presence of CD44 marker (control vs modified hBM-MSCs p <0.05) (Figure 5B). At the same time, 48.33 ± 10.5% of cells transfected with mRNA-ITGA4 and 38.22 ± 8.61% of control cells were found to co-localize ED1 antigen, typical for activated microglia and macrophages (Figure 5C). These results indicate that 72 hours after transplantation, more genetically modified cells in comparison to naive hBM-MSCs were phagocytosed by the recipient's immune cells.

### Homogenate from injured brain repels hBM-MSC

**Figure 6:**
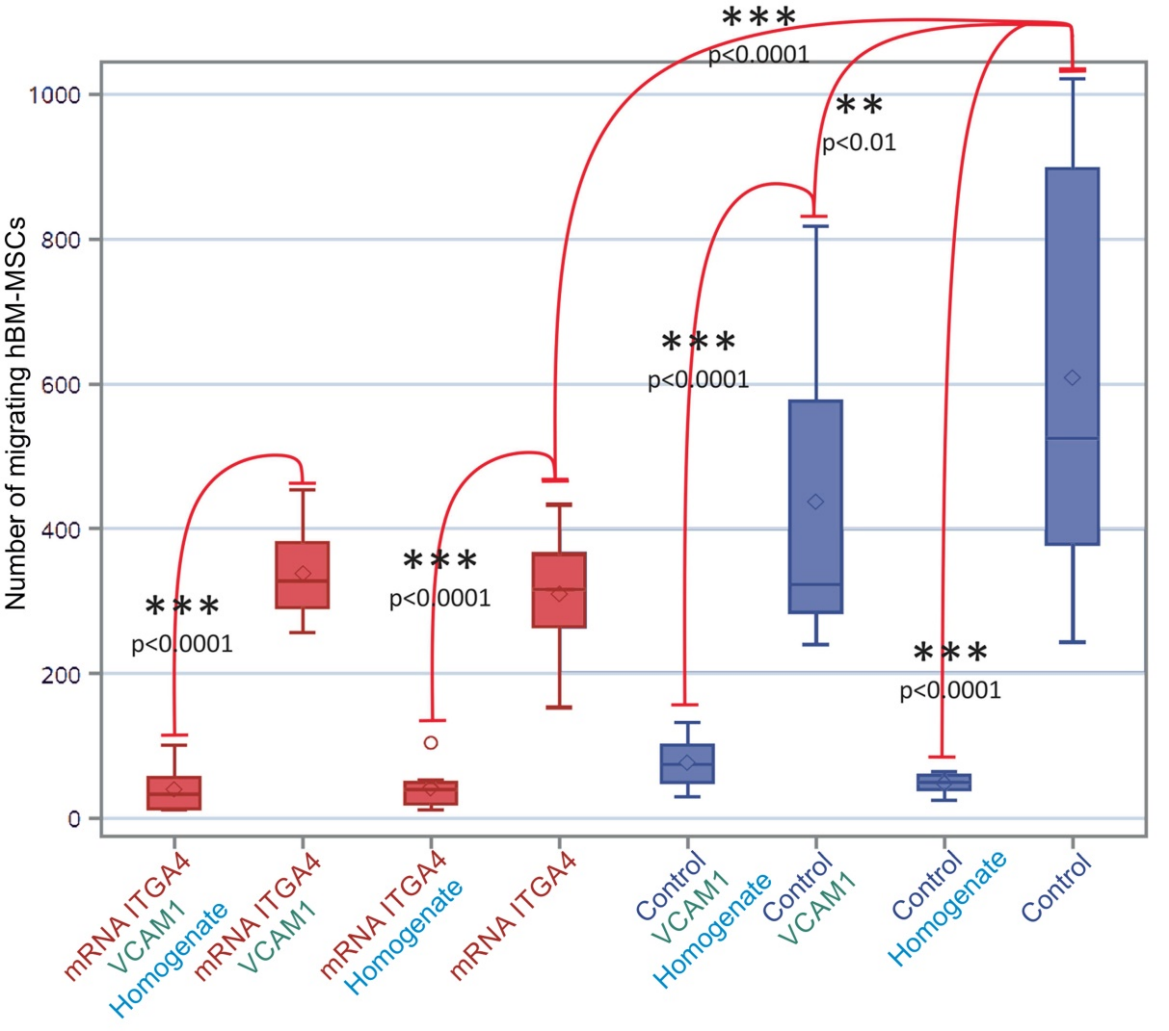
The number of mRNA-ITGA4 transfected (red boxes) and Control (violet boxes) hBM-MSCs migrating toward injured brain homogenate during 24 hours (transwell chambers assay). Homogenate from injured brain strongly repelled hBM-MSCs regardless of cell engineering or insert coating. The type III fixed effect test was used to determine statistical significance and the LMS method was used to compare. Box charts present the dispersion and shape of the data distribution for the test value in both tested cell populations. The length of the bars is equal to the quarter range (Q1-Q3) of the data, the tips of the moustaches indicate the minimum and maximum values, the line inside of the bar determines the median, while the square the arithmetic mean, the outliers are presented in the form of circles. *p<0.05, **p<0.01, ***p<0.001 (n=6). mRNA-ITGA4 - hBM-MSCs transfected with mRNA-ITGA4; Control - naive hBM-MSCs; Homogenate - homogenate of the brain tissue isolated from rats subjected to focal brain injury insult placed in the lower chamber of the transwell; VCAM1 - the membrane inserted between the upper and the lower transwell chambers, covered with VCAM1 protein.

The mRNA-ITGA4 transfected hBM-MSCs placed onto collagen-coated inserts were characterized by lower spontaneous migration to the bottom surface of the membrane in comparison to naïve hBM-MSCs (p˂0.0001). The additional coating of an insert with VCAM1 protein decreased the migration of naïve hBM-MSCs (p˂0.01), while it did not have any impact on mRNA-ITGA4 transfected hBM-MSCs. It is interesting to note that the addition of homogenate from injured brain to the lower compartment of the chamber strongly inhibited hBM-MSCs migration of both naïve and mRNA-ITGA4 transfected cells regardless of type of insert coating (p˂0.0001) (Figure 6).

## Discussion

We have shown that intra-arterially injected MSCs locate in the perivascular space of rat brains subjected to focal brain injury. Intriguingly, homogenate from injured brain was repulsive against hBM-MSCs in transwell experiments, which is in striking contrast to its attractive force toward NSCs reported in our previous article 20. It can also explain a lack of migration by MSCs towards deeper brain parenchyma observed in our current study. Scientists have been aware for several years that the main benefits of MSC transplantation in the post-stroke recovery process results from immunomodulation, neuroprotection and pro-angiogenic activity 1,21,22. Our work indicates that biological activity of transplanted MSCs also may be exerted through modulation of the neurovascular unit, which is in part composed of endothelial progenitor cells (EPC) and pericytes. EPC transplantation has also been shown to improve regeneration after stroke 23. Grafted EPCs provided improvement to micro-vessels' integrity, reduction of perivascular edema and BBB sealing. Both EPC and MSCs were also shown to be able to perform mitochondria transfer to damaged cells and to regulate neuroinflammation 24,25. These mechanisms are common for both mentioned cell types, may be exerted from vascular niche and are clinically important contributors in post-stroke recovery, which directly translates into functional outcome 22,26. Pericytes are another important cell type involved in regeneration of vessel structure and function. Perivascular niche is a natural location of pericytes, and this cell type has been recently linked to MSCs 27**.**

Moreover, pericytes play a pivotal role in regulation and maintenance of the neurovascular unit, including matching metabolic demand of neurons with cerebral blood flow, persistence of blood brain barrier (BBB), control of leukocyte trafficking, and angiogenesis 28. Importantly, pericytes are highly vulnerable to brain injury and their dysfunction has a profound role in ischemic pathology 29. It has been shown in a 3D co-culture model that MSCs match the performance of pericytes in increasing trans-endothelial electric resistance and inhibiting permeability of macromolecules 30. On the other hand, MSCs have been derived from the perivascular space 31. Therefore, transplanted MSCs may contribute to pericyte re-population and restoration of ischemia-induced malfunction of the neurovascular unit, which in turn may facilitate neuroregeneration and improvement of the CNS function. A fascinating phenomenon was observed in a recent *in vitro* study in which MSCs placed in the upper compartment of a transwell plate formed a long filopodia directed to cortical neuron cultured after oxygen glucose deprivation procedure in the lower compartment. Through this type of long-distance interaction, MSCs were able to improve survival of injured neurons. Moreover the same authors were able to show that intra-arterially transplanted commercially available MSCs in MCAO stroke model were able to both reduce the infract region and neurological deficit in rats 32. This data has led FDA to approval of new clinical trials for stroke with MSC transplantation. The repulsive activity of injured CNS tissue may keep MSCs in the perivascular niche and protect them from the hostile post-ischemic tissue environment, whereas other indirect contact surrogate mechanisms ensuring neuroprotection such as contact via filopodia and paracrine activity may be still exerted.

While there is certainly a potential for MSCs to contribute to neurorepair, many obstacles need to be overcome before MSCs may be introduced to the clinic 33. In most previous clinical trials as well as the majority of animal studies, MSCs were transplanted intravenously in tremendously huge doses to achieve clinical efficacy. Infused cells spread through the host's entire body and only a negligible number of them homed to the brain. This was connected with the high risk of general induction of thrombus formation and pulmonary embolism 34. Some scientists implemented remedies in the form of co-administration of anticoagulants such as heparin, which contributed to improvement of therapy outcome 35,36. In case of IA cell administration, a much lower cell dose is necessary to obtain a significantly higher cell settlement in the brain. Cells do not pass through lung vasculature, eliminating pulmonary embolism risk. The use of heparin is in opposition to our initial approach of transplanted cell adhesion enhancement, thus we could not implement anticoagulants in our study. However, our group is aware of the risk of micro-embolism development after IA transplantation of MSCs 37. In our previous study we investigated this issue and showed that proper infusion speed and cell number adjustment are crucial to avoid post-transplantation complications such as micro-stroke 37,38. These experimental settings were implemented in our current work to ensure the safety of MSC transplantation.

Although relatively high in comparison to IV infusion, brain engraftment rate is still low in IA injection, and this might be a factor limiting the size of the therapeutic effect. While improved docking of VLA-4 engineered GRPs translated into effective diapedesis, we have not observed such an effect for MSCs. This might stem from a radically different response to injured CNS tissue. CNS injury-based chemoattraction can easily drag endothelium-adhering neural stem cells across the vessel wall, while the same factors repel MSCs, which might be a reason for the thorough clearance of MSCs. However, MSCs still are present for many hours in the cerebral vasculature, and they may convey many therapeutic actions, including the release of EVs and neurotrophic factors 22,39. As we have shown previously, the EVs secreted by hBM-MSCs can provide a similar immunomodulatory effect as the cells of their origin after IA transplantation in a focal brain injury model 40**.** Thus it is possible that higher mRNA-ITGA4 transfected hBM-MSC accumulation over the time period of 24 hours in blood vessels, which we have obtained, is sufficient to improve recovery due to potentially higher paracrine activity of transplanted cells over a prolonged period of time. In addition, there seems to be a sub-population of rare cells among the population of transplanted MSCs capable of engrafting and locating in the perivascular space, which is certainly worthy of further study for their enrichment in a population of transplanted cells to boost therapeutic effect.

The adhesion molecule-dependent process of diapedesis described in leukocytes has been long recognized 41. It is a relatively fast process within a time-frame of minutes or hours during which cells are actively crossing the vessel wall, and it was observed in our previous work with adhesion molecule-engineered GRPs 10. Here, the extravasation of MSCs is observed over a period of three days, therefore we might be dealing with another process entirely. Additionally, the mRNA-based production of ITGA4 protein lasts up to 24 hours, therefore it cannot be involved in cell migration across the vessel wall.

ITGA4 engineered cells were more vulnerable to phagocytosis, probably due to transfection-related toxicity, therefore cell engineering does not seem to affect their extravasation. According to the literature, exogenous mRNA introduction may result in elevation of pro-inflammatory cytokine synthesis, which increases the likelihood of host immune cell activation toward transplanted modified cells 42. Thus, a higher percentage of ED1 positive cells after mRNA-ITGA4 modified hBM-MSCs injection observed in our experiments could be due to the introduction of exogenous mRNA to MSCs. The degree of exogenous mRNA immunogenicity is closely related to the type of chemical modification the molecule has undergone 42. Further optimization of mRNA molecules is necessary before MSC transfection and transplantation.

Of note, a recent study with the use of intravital microscopy of zebrafish revealed a distinct process of MSC extravasation called angiopellosis, in which cells are relatively passive, while the vascular wall undergoes remodelling to absorb MSCs 43. Taking into consideration the long time-frame of MSC extravasation in our study and independence from induced expression of adhesion molecules, we speculate that angiopellosis might be responsible for this process and it warrants further studies with intravital microscopy to demonstrate if angiopellosis is a phenomenon only observed in fish, or it plays an essential role in stem cell trafficking across a variety of species.

Our study has several limitations. The observation time was limited to 3 days post-transplantation, and longer observation, preferable with the application of intravital microscopy, is warranted to gain deeper insight into the long-term fate of transplanted cells. Also, we used human cells to maximize the translational value of transplanted cells but a xeno-barrier may affect cellular behaviour and the same cells transplanted to the human brain may perform differently. We argue that using rat MSCs is not a solution to this since there is a profound difference in the behaviour of rodent and human MSCs. Also, we did not knock out ITGB1 in MSCs to get deeper insight into the role of this molecule, but overall the role of adhesion molecules in MSC extravasation might be marginal and the relatively unexplored mechanism of angiopellosis may play a more dominant role.

## Conclusions

In conclusion, intra-arterially injected MSCs extravasate to localize in the perivascular space, a natural niche for pericytes, which play a pivotal role in regulating and maintaining the neurovascular unit. Since pericytes are highly vulnerable to ischemia, their replacement by MSCs transplantation might be a viable approach. However, the engraftment rate of MSCs is very low, therefore currently this mechanism is not playing a dominant therapeutic role. More probably, the prolonged presence of MSCs in the lumen of cerebral vasculature is responsible for the therapeutic effects widely described in the literature. Our mRNA-ITGA4 transfection method is an efficient way to improve the initial settlement of MSCs in the injury area, however it apparently does not translate into their diapedesis. Since mRNA-ITGA4 expression lasts just a few hours after transplantation, it is not surprising that both engineered and naïve MSCs are cleared at the same pace until a certain number of cells equal for both cell populations is anchored in the cerebral vasculature. On the third day, we observed extravasation of nearly half of cells present at that time in the cerebral vasculature to the perivascular space with no statistical difference between mRNA-ITGA4 transfected and naive hBM-MSCs. However, previously engineered cells have proven to be more vulnerable to host immune attack, which might be related to induction of cell immune response and their marking for destruction. Induction of ITGA4 expression seemed not to have played an important role in MSC extravasation, which instead might be executed through angiopellosis. The natural location of extravasated MSCs in the perivascular space is very promising and further studies on recruitment of a higher number of transplanted MSCs are particularly compelling. In summary, MSCs are attractive as a therapeutic agent for treatment of focal brain injuries, and further research may help fully exploit their therapeutic potential.

## Supplementary Material

Supplementary figures and tables.

Video FigS1control.

Video FigS1transfected.

Video FigS3control.

Video FigS3transfected.

## Abbreviations

ARCA: anti-reverse-cap-analogue
BBB: blood brain barrier
BSA: bovine serum albumin
CCA: common carotid artery
CNS: central nervous system
ECA: external carotid artery
EVs: extracellular vesicles
GRPs: glial restricted precursors
hBM-MSCs: human bone marrow-derived MSCs
IA: intra-arterial
ICA: internal carotid artery
IHC: immunohistochemistry
ITGB1: integrin beta-1
LMS method: last mean square
MCGS: mesenchymal cell growth supplement
Molday ION: SPIO conjugated with rhodamine B
MR: magnetic resonance
MRI: MR imaging
mRNA-ITGA4: mRNA coding integrin α4
MSCBM medium: mesenchymal stem cell growth medium
MSCs: mesenchymal stem cells
NSCs: neural stem cells
PBS: phosphate-buffered saline
PFA: paraformaldehyde
SPIO: superparamagnetic iron oxide nanoparticles
VCAM1: vascular cell adhesion molecule-1
VLA-4: integrin α4β1
EPC: endothelial progenitor cells
