# Supplementary material for: Mesenchymal stem cells injected into carotid artery to target focal brain injury home to perivascular space
Source: Theranostics. 2020 May 17;10(15):6615–28. doi: 10.7150/thno.43169 (PMC7295043; doi:10.7150/thno.43169)
Supplement: Supplementary file 1 — Supplementary figures and tables. [file thnov10p6615s1.pdf]

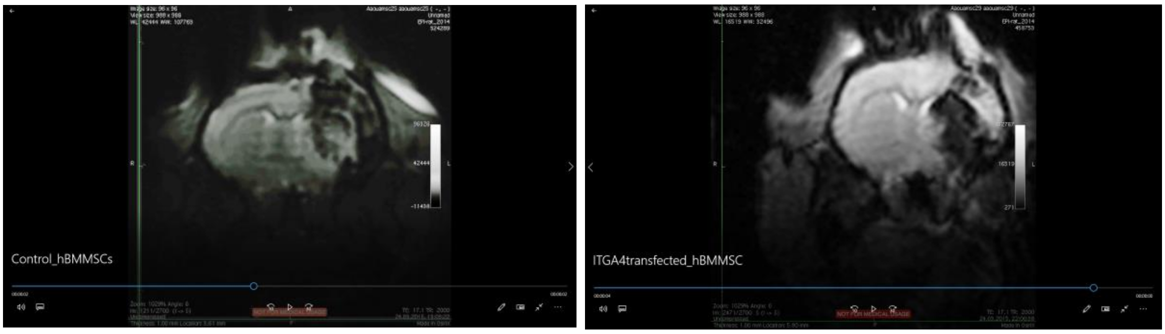

Figure S1. Real-time MRI influx recordings of IA injected mRNA-ITGA4 transfected or control (naïve) hBM-MSCs into the rat brain subjected to focal brain damage. Transplanted cells (visible as hypointensive signal due to Molday ION labelling) have arrived directly in the right hemisphere and localized within infarcted area (visible as hyperintensive region) including striatum and cortex.

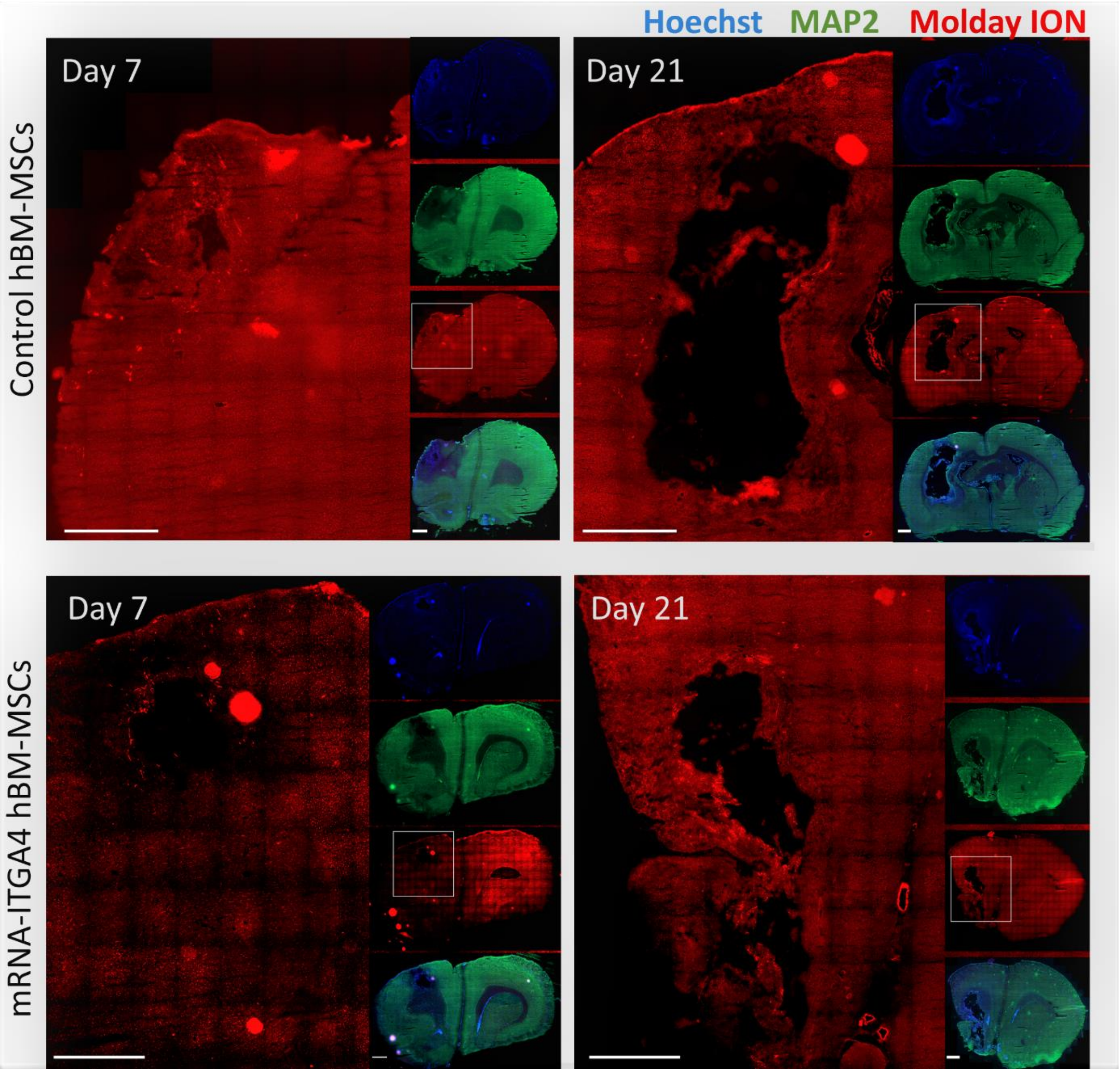

Figure S2. Tile scans of immunohistochemically stained brain of rats subjected to the focal brain injury. The analysis of the presence of IA transplanted hBM-MSCs in the different cross section planes have been examined on the seventh and twenty-first days after cell transplantation. No mRNA-ITGA4 hBM-MSCs neither their naïve counterparts (red) were seen in most brain tissue cross-sections. The MAP2 antibody staining (green) was used to display the necrotic penumbra area. The cell nuclei were counterstained with Hoechst 33258 (blue). Scale 1mm.

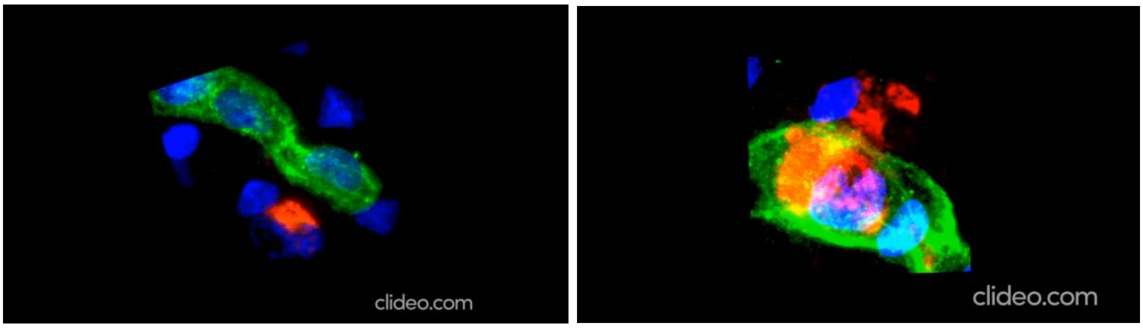

Figure S3. 3D projection of immunohistochemical analysis of transplanted hBM-MSCs located within the area of focal brain injury in relation the vessel lumen through on third day after IA transplantation in rats with focal brain injury. mRNA-ITGA4 transfected and naïve hBM-MSCs were visible outside blood vessels lumen on day 3 after IA injection. Both populations of hBM-MSCs were labelled with Molday ION (red). Endothelial cells were visualized using antibody against Claudine 5 (green). The cell nuclei were counterstained with Hoechst 33258 (blue).
